# Practitioners' Bookshelf

**Published:** 1892-07-23

**Authors:** 


### PRACTITIONERS' BOOKSHELF.

### HYGIENE AND SANITARY SCIENCE.*

Dr. Wilson's book need s no introduction to the medical profession, for a work which has run through seven editions in a comparatively short period has already proved its value. Yet we cannot omit to notice some of the features which have been introduced into the latest edition, and which show that the author is determined to be thoroughly up to date in treating of a subject which has become a science of itself, and which is progressing almost every day in its range and importance. His minute revision of the whole work shows also that his aim is that the matter shall be presented in each edition in the most acceptable form. It is indeed a practical work of reference for the medical man and a guide to the sanitary officer. It has long been among the very best of the smaller works on the subjects, and the numerous additions, amounting to 200 pages in this edition, have made it probably the most complete handbook we possess. Indeed, the difficulty is what to exclude. But Dr. Wilson has not been tempted to sacrifice clearness to a desire to drag in theories which are unproved and uncertain in the present state of science. In every sense the work is a practical one. For the sanitary officer he gives digests of no less than eighteen of the recent Public Health Acts, including the London Act of 1891; and in appendices we get not only a valuable collection of formulæ and data, such as that of Hurst, for the velocity and discharge of egg-shaped sewers, with those of Toricelli, Boyle, and Hughes, and examples of their application, but also a list of examination questions with their solutions. The chapters on the meat of diseased animals and the microbes present in infectious diseases demanded special care and judgment; and we find here accounts of the most recent epidemics, and reference to some of the best reports on the subjects. Dr. Wilson insists that all milk from tuberculous animals should be condemned, and quotes the Glasgow decision as to their flesh, though we should like to see here a fuller statement upon this difficult question. He does not fail to notice the theoretical inefficiency of sulphur fumigation, regarding it as only an aid to practical disinfection; and, in opposition to the common inefficient vaccination, he draws attention to the importance of the number and quality of the cicatrices. It is interesting to note that after all has been said in favour of cremation he cannot discover that earth-burial properly carried out has ever of itself been productive of a well authenticated outbreak of specific disease. The real danger lies in the retention of dead bodies among the living before burial, and the neglect of precautions, than against infectious disease. The revision of the chapters on the analysis of air and water, and that on sewage disposal, has involved much pains and labour, and the accounts given of recent outbreaks of infectious disease are models of clear, succinct description. The more we examine the work the more we are impressed with the amount and exactness of the information conveyed, and the wide range of the subjects treated. There is no text-book of the size which can be more thoroughly recommended.

* "Handbook of Hygiene and Sanitary Science." By George Wilson, M.A., M.D., F.R.S., Ed. D.P.H. Cantab. Seventh Edition, 8vo., pp. 750. Illustrated. (London: J. and A. Churchill, 1892.)

